# Association between conscientiousness and team emotional intelligence: A moderated mediation model

**DOI:** 10.1097/MD.0000000000031001

**Published:** 2022-10-21

**Authors:** Xuefei Zhou, Xueqin Sun, Zhao Wang, Tao Jiang

**Affiliations:** a The First Affiliated Hospital of Bengbu Medical College, Bengbu City, China; b Office of Academic Affairs, Anhui University of Science and Technology, Bengbu City, China; c School of Foreign Languages, Anhui University of Finance and Economics, Bengbu City, China.

**Keywords:** conscientiousness, emotional intelligence, interpersonal emotion regulation, positive emotional climate interaction, team emotional intelligence

## Abstract

To investigate the influence of interpersonal emotion regulation and conscientiousness on team emotional intelligence. A total of 1369 college students were investigated with the conscientiousness subscale of Big Five Personality Questionnaire, Team Emotional Intelligence Scale and Leadership Positive Emotional Operation Questionnaire. Variance analysis, Pearson product difference correlation analysis, multiple regression analysis and path analysis were used. In order to avoid the possible skew problem, the bootstrap method was used to calculate the structural equation model. SPSS 22.0, Amos 24, *R* software were used for statistical analysis. A total of 1600 questionnaires were sent out and 1369 effective questionnaires were recovered. The total score of College Students’ team emotional intelligence was 5.07 ± 0.70, with 4.88 ± 0.87, 5.38 ± 0.79, 4.74 ± 0.91, 4.71 ± 0.83, 5.23 ± 1.00, and 5.46 ± 0.91 for interpersonal understanding, asking for feedback, emotional management, organizational cognition, relationship building and problem-solving ability, respectively. Conscientiousness significantly predicted team emotional intelligence, and leadership’s positive emotional operation. Furthermore, conscientiousness could predict team emotional intelligence through mediating individual emotional intelligence. Interpersonal positive emotion regulation played a part of mediating role between conscientiousness and team emotional intelligence.

## 1. Introduction

Team is an organization composed of two or more individuals who interact and depend on each other. Recently, teams exist in a wide range of work and learning units, such as psychological counseling, students’ learning, and scientific research team. Previous studies have shown that teams good at dealing with member relationships are more likely to achieve high performance than those with members of outstanding skills and high intelligence.^[1]^ Druskat defines the team’s ability to deal with member relationships as team emotional intelligence, which reflects the team’s ability to perceive, evaluate, adjust and express various emotional information, and interact with other teams.^[2]^ Team emotional intelligence can not only improve cooperation level and learning capbility,^[3]^ and increase team satisfaction, trust and cohesion,^[4]^ team performance,^[5,6]^ and team creativity,^[7,8]^ but also reduce competitive behavior.^[9]^

Recent research confirmed that Big Five personality, including conscientiousness, extroversion, agreeableness and emotional stability were closely related to team process and team results.^[10]^ In addition, previous studies have found that conscientiousness was the most important predictor among the five factors of personality.^[11,12]^ However, considering that personality traits were relatively stable, it was necessary to explore the intermediate variables between conscientiousness and team emotional intelligence.

Individual emotional intelligence has an important influence on the formation of team emotional intelligence. Chernis^[13]^ proposed model of emotional intelligence and organizational effectiveness, emphasizing that individual emotional intelligence could change team level emotional intelligence. Therefore, individual emotional intelligence may play a mediating role between conscientiousness and team emotional intelligence. Furthermore, social interaction theory finds that employees with low conscientiousness are more vulnerable to the impact of external environment.^[14]^ When leaders have lower positive emotional interaction, members perceive less organizational support, which will reduce team communication, and hinder the improvement of team emotions. Therefore, positive emotional interaction of leaders may play a regulatory role between conscientiousness and team emotional intelligence.

The relationship among conscientiousness, individual emotional intelligence, leader’s positive emotional interaction and team’s emotional intelligence should be complex, and needs to be further investigated. This study attempts to analyze the influencing factors and mechanism of emotional intelligence of the team through establishing a theoretical model to enhance team emotional intelligence and guide team management.

## 2. Methods

### 2.1. Research objects

In this study, stratified random cluster sampling method was used, first stratified by grade, then randomly selected classes. A total of 1369 nursing college students from three universities in Anhui Province were investigated, including 403 male students (29.40%) and 966 female students (70.60%) with an age of 21.52 ± 1.42.

### 2.2. Tools and methods

#### 2.2.1. Responsibility subscale of big five personality questionnaire^[15]^

The scale was compiled by Goldberg (1992) on the basis of big five personality theory and used to measure five dimensions of personality: neuroticism, extraversion, openness, agreeableness, and conscientiousness. There are 10 items in the accountability subscale, among which 15, 30, and 55 are reverse scoring questions. The higher the score, the more conscientious the respondents were. In this study, Cronbach’s coefficient is 0.772.

#### 2.2.2. Team emotional intelligence questionnaire^[16]^

The team emotional intelligence scale developed by Hamme is used to measure the ability of team emotional perception, evaluation, expression and regulation. The scale has a good validity, which is verified in the samples of students, military and employees in different organizations. There are 28 projects, including six dimensions: interpersonal understanding, feedback solicitation, emotional management, organizational cognition, relationship building, and problem solving. In this study, Cronbach’s α coefficients were 0.904, 0.875, 0.722, 0.597, 0.792, and 0.737, respectively. The α coefficients of Cronbach’s off the total table were 0.955.

#### 2.2.3. Emotional intelligence questionnaire^[17]^

The scale was compiled by Wong & Law to measure individual level emotional intelligence, which is widely used in China. The scale has 4 dimensions and 16 items, namely self-emotion perception, emotion control, emotion utilization and emotion perception of others. The questionnaire uses Likert 5-point score, with high score representing better emotional intelligence. Cronbach’s α coefficients are 0.814, 0.845, 0.789, and 0.874, respectively. The α coefficient of the total table is 0.891.

#### 2.2.4. Leadership positive emotion interaction questionnaire^[18]^

Ozcelik’s questionnaire was used to measure leaders’ emotional mobilization and support to team members. The scale has a good structure validity, which has been verified in the samples of students and employees in different organizations. There are 6 items in the questionnaire, and Likert grade 7 score is used. Cronbach’s α coefficient is 0.919.

### 2.3. Measurement process

After obtaining the students’ informed consent, the group test was carried out in class. The questionnaire was issued in December 2018. Before the survey, it was explained to the subjects that the content of the questionnaire would be strictly confidential. The results of the questionnaire were only used for scientific research, and the subjects were required to answer carefully and independently according to the guidance. It will take about 15 minutes for the subjects to complete all the questionnaires, and all the questionnaires will be collected on the spot. In this study, 1600 questionnaires were sent out and 1369 effective questionnaires were recovered, with a recovery rate of 85.56%.

### 2.4. Statistical methods

SPSS 22.0, Amos 24, *R* software were used for statistical analysis. Variance analysis, Pearson product difference correlation analysis, multiple regression analysis and path analysis were used. In order to avoid the possible skew problem, the bootstrap method was used to calculate the structural equation model.

## 3. Results

### 3.1. Common method deviation inspection

Harman single factor test was used to test the common method deviation of all items in the related questionnaire.^[19]^ The results showed that 46 factors with eigenvalue greater than 1 were used in the non-rotating exploratory factor analysis, and the first factor explained the variance as 19.23%, which was less than the critical standard of 40%, indicating that the common method deviation in this study was not obvious.

### 3.2. Descriptive statistics and correlation analysis

The total score of College Students’ team emotional intelligence was 5.07 ± 0.70, with 4.88 ± 0.87, 5.38 ± 0.79, 4.74 ± 0.91, 4.71 ± 0.83, 5.23 ± 1.00, and 5.46 ± 0.91 for interpersonal understanding, asking for feedback, emotional management, organizational cognition, relationship building and problem-solving ability, respectively. There were significant differences in age, relationship with classmates, teachers, parents and family atmosphere. The results of descriptive statistics and correlation analysis were shown in Table 1. Conscientiousness was positively correlated with individual emotional intelligence, leadership positive emotional interaction, and team emotional intelligence.

**Table 1:** Descriptive statistics of each variable and person correlation coefficient (n = 1369).

Variables	$\bar{x}\pm s$	1	2	3
Conscientiousness	3.594 ± 0.436	1		
Team emotional intelligence	5.050 ± 0.749	0.340**		
Emotional intelligence	4.039 ± 0.728	0.474**	0.409**	
Positive emotional climate interaction	5.386 ± 0.900	0.294**	0.683**	0.371**

*P < .05.

** *P* < .01.

****P < .001.

### 3.3. Mediating effect of individual emotional intelligence between conscientiousness and team emotional intelligence

**Figure 1.**
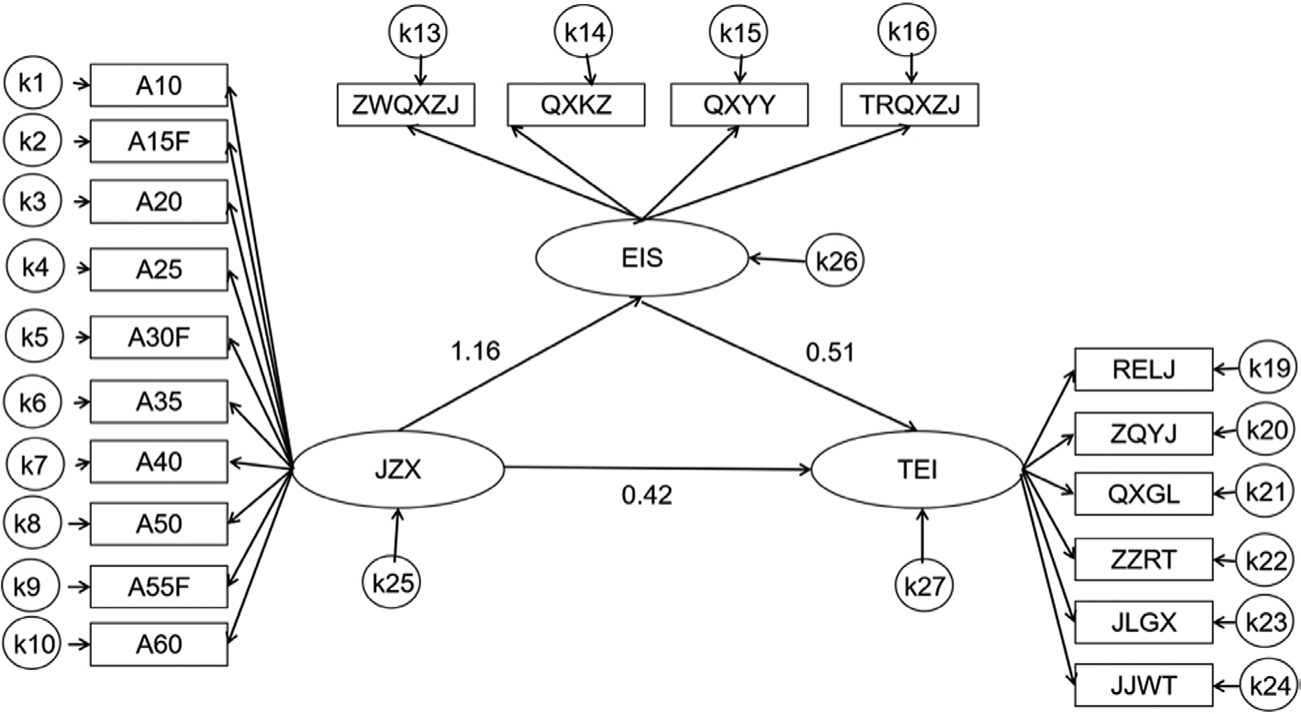
The mediating role of individual emotional intelligence between conscientiousness and team emotional intelligence. EIS = individual emotional intelligence, JJWT = problem solving, JLGX = relationship building, JZX = conscientiousness, QXGL = emotion management, QXKZ = emotion control, QXYY = emotion application, RELJ = interpersonal understanding, TEI = team emotional intelligence, TRQXZJ = emotion perception of others, ZQYJ = feedback seeking, ZWQXZJ = self emotion perception, ZZRT = organizational cognition.

Amos 24.0 software was used to establish the mediating effect model between personal emotional intelligence and team emotional intelligence according to the research hypothesis. The fitting indexes were as follows: Comparative Fit Index (CFI) = 0.944, Tucker–Lewis Index (TLI) = 0.935, Incremental Fit Index (IFI) = 0.944, Relative Fit Index (RFI) = 0.918, Normal Fit Index (NFI) = 0.929, Root Mean Square Error of Approximation (RMSEA) = 0.049, indicating that the model fits well, as shown in Figure 1. The path values were shown in Table 2. Bootstrap method was used to test the significance of intermediary effect, with 5000 bootstrap samples randomly selected to fit the model. The results showed that the total effect was 0.434, in which the direct effect was 0.180 (95% CI: 0.082, 0.276; *P* < .001), and the indirect effect was 0.255 (95% CI: 0.185, 0.332; *P* < .001). The results indicated that personal emotional intelligence played an intermediary role between conscientiousness and team emotional intelligence.

**Table 2:** The mediating role of individual emotional intelligence between conscientiousness and team emotional intelligence.

Dependent variables		Independent variables	β	SE	CR	*P*
EIS	<---	JZX	1.16	0.07	17.82	<.001
TEI	<---	EIS	0.51	0.07	7.26	<.001
TEI	<---	JZX	0.42	0.12	3.60	<.001

EIS = individual emotional intelligence, JZX = conscientiousness, TEI = team emotional intelligence.

### 3.4. Mediating effect between individual emotional intelligence and leader’s positive emotional interaction

**Figure 2.**
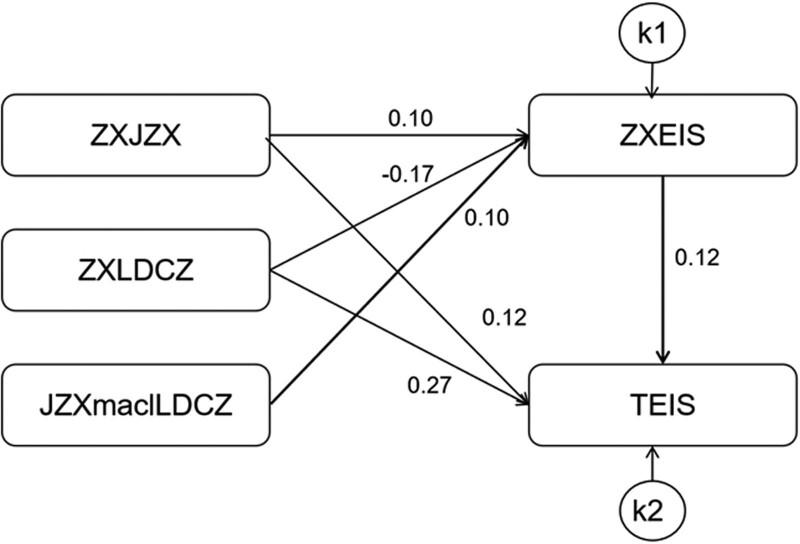
The first half path regulation effect of leader’s positive emotional interaction between conscientiousness and team’s emotional intelligence. JZXmadlLDCZ = product of conscientiousness and leadership positive emotional operation, TEIS = team emotional intelligence, ZXEIS = centralized personal emotional intelligence, ZXJZX = centralized conscientiousness, ZXLDCZ = centralized leadership positive emotional operation.

Amos 24.0 software was used to establish a regulatory model of leadership initiative in the first half of the path of conscientiousness, individual emotional intelligence and team emotional intelligence according to the research hypothesis. The fitting indexes were as follows: CFI = 0.999, TLI = 0.998, IFI = 0.999, RFI = 0.997, NFI = 0.999, RMSEA = 0.031, indicating that the model fits well, as shown in Figure 2. The values of each path were shown in Table 3.

**Table 3:** The moderating effect of leadership positive emotional interaction in the first half of the path between conscientiousness and team emotional intelligence.

Dependent variables		Independent variables	β	SE	CR	*P*
EIS	<---	ZXZJX	0.10	0.01	16.91	<.001
EIS	<---	ZXLDCZ	−0.17	0.03	-5.01	<.001
EIS	<---	JZXmalLDCZ	0.10	0.01	16.91	<.001
TEIS	<---	EIS	0.12	0.02	6.03	<.001
TEIS	<---	ZXZJX	0.12	0.02	6.03	<.001
TEIS	<---	ZXLDCZ	0.27	0.02	12.22	<.001

EIS = individual emotional intelligence, JZXmadlLDCZ = product of conscientiousness and leadership positive emotional operation, TEIS = team emotional intelligence, ZXJZX = centralized conscientiousness, ZXLDCZ = centralized leadership positive emotional operation.

### 3.5. Direct path regulation effect of leader positive emotional interaction between conscientiousness and team emotional intelligence

**Figure 3.**
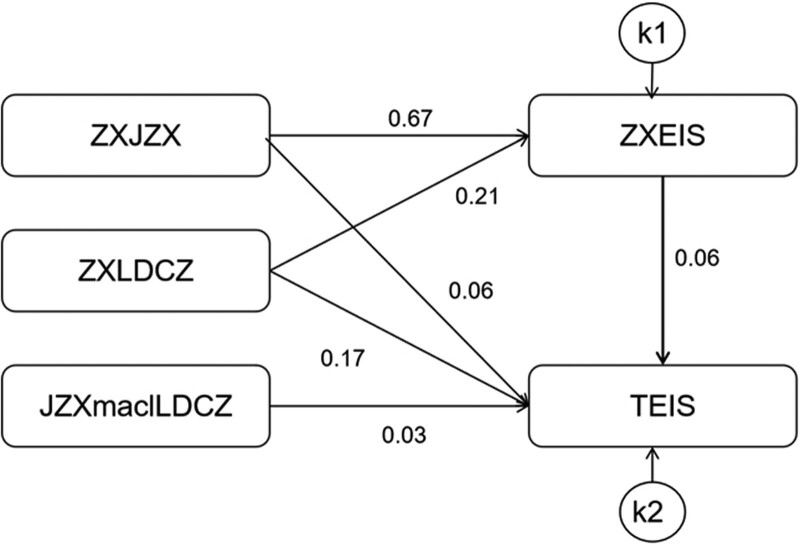
A test of the direct path regulation effect of leader’s positive emotional interaction between conscientiousness and team’s emotional intelligence. JZXmadlLDCZ = product of conscientiousness and leadership positive emotional operation, TEIS = team emotional intelligence, ZXEIS = centralized personal emotional intelligence, ZXJZX = centralized conscientiousness, ZXLDCZ = centralized leadership positive emotional operation.

The fitting indexes for the model of the direct path regulation effect of leader positive emotional interaction between conscientiousness and team emotional intelligence were as follows: CFI = 0.999, TLI = 0.993, IFI = 0.999, RFI = 0.992, NFI = 0.998, RMSEA = 0.064. The results indicated that the model fitted well, as shown in Figure 3. The path values were shown in Table 4.

**Table 4:** The direct path moderating effect of leadership positive emotional interaction between conscientiousness and team emotional intelligence.

Dependent variables		Independent variables	β	SE	CR	*P*
ZXEIS	<---	ZXZJX	0.67	0.04	16.70	<.001
ZXEIS	<---	ZXLDCZ	0.21	0.02	10.61	<.001
TEIS	<---	ZXEIS	0.06	0.03	2.18	.029
TEIS	<---	ZXZJX	0.06	0.03	2.18	.029
TEIS	<---	ZXLDCZ	0.17	0.05	3.46	<.001
TEIS	<---	JZXmalLDCZ	0.03	0.01	2.56	.010

JZXmadlLDCZ = product of conscientiousness and leadership positive emotional operation, TEIS = team emotional intelligence, ZXEIS = centralized personal emotional intelligence, ZXEIS = centralized personal emotional intelligence, ZXJZX = centralized conscientiousness, ZXLDCZ = centralized leadership positive emotional operation.

## 4. Discussion

This study used college students as subjects, personal emotions as intermediary variables, and leadership positive emotions as moderating variables, to evaluate the influence of conscientious personality on team emotional intelligence through building a moderated intermediary model.

The results showed that conscientiousness could predict team emotional intelligence through mediating individual emotional intelligence, which was similar to the previous research.^[10,14,20,21]^ According to the emotional intelligence cascade model proposed by Joseph, conscientiousness can predict emotional perception, emotional use and emotional understanding.^[20]^ Nawi and other studies found that people with high conscientiousness scores have more self-discipline and emotional self-regulation ability. There were two possible reasons for the influence of individual emotional intelligence on team emotional intelligence. First, according to the emotional intelligence and organizational effectiveness model, individual emotional intelligence can change team emotional intelligence. Highly responsible members are task-oriented and self-motivated. When the team’s average level of responsibility is higher, members are more willing to communicate with others, actively express their own views, and invest more emotional commitment in the team.^[22]^ Second, leaders with high responsibility encouraged themselves and others to develop a higher sense of responsibility.^[23]^ Therefore, conscientiousness can affect individual emotional intelligence, and then contribute to team emotional intelligence.

Positive emotional interaction of leadership could play a regulatory role not only in the relationship between conscientiousness and team emotional intelligence, but also in the intermediary chain of “conscientiousness individual emotional intelligence team emotional intelligence”. Specifically, team with high positive emotional interaction could be better predicted by conscientiousness. The results showed that there were individual differences in the effect of conscientiousness on team emotional intelligence. The positive emotional interaction of leaders weakened the effect of conscientiousness on team emotional intelligence. In the team with low positive emotional interaction, the effect of conscientiousness on team emotional intelligence was reduced. When team members feel a high degree of external support, it will enhance their sense of belonging and identity to the organization, stimulate their sense of ownership, increase communication within the team, and create a good organizational atmosphere, which will further promote the improvement of team emotional intelligence. The positive emotional interaction of leaders plays a compensatory role in the conscientiousness. Moreover, the results showed that positive emotional interaction of leaders could regulate the influence of other variables on individual emotional intelligence. There are at least two mechanisms in the process of leading positive emotional interaction on emotional intelligence.^[23]^ First, the emotion inducing mechanism, that the leader’s behavior style, as an external stimulus, can directly induce team members to produce specific emotional feeling and improve their emotional perception ability. Second, the emotional contagion mechanism, that the leader’s emotions directly lead the members to experience similar emotions through the contagion process. Therefore, conscientiousness is more likely to enhance team emotional intelligence through improved performance of the individual emotional intelligence.

### 4.1. Strength and limitation

The moderated mediating model not only evaluate the effects of conscientiousness on the formation of team emotional intelligence, but also explore the mediating effect of individual emotional intelligence, which may deepen and expand the understanding of intermediate variables for conscientiousness and team emotional intelligence. In addition, some implications from the moderated mediation model may be applicable to improve the emotional intelligence of the team. First, members with high responsibility can be recruited. Second, when the team members are fixed, leaders can strengthen the training of positive emotional interaction, which will be compensate for a low conscientiousness.

There are also several limitations in this study. First, the results are only based on the self-report of college students. Study with more objective methods to collect data is needed in the future. Second, the cross-sectional design is adopted in this study. Evidence with follow-up design is still needed to verify the findings. Third, this study has included participates from only one province. Further study including the participates from different background is still needed.

Finally, this study only examined the positive emotional interaction in interpersonal emotion regulation, with no consideration of possible negative emotional interaction.

In conclusion, interpersonal positive emotion regulation plays a part of mediating role between conscientiousness and team emotional intelligence.

## Author contributions

**Conceptualization:** Xuefei Zhou, Xueqin Sun.

**Data curation:** Xuefei Zhou.

**Formal analysis:** Xueqin Sun.

**Investigation:** Tao Jiang.

**Methodology:** Tao Jiang.

**Resources:** Xuefei Zhou.

**Writing – original draft:** Zhao Wang.

**Writing – review & editing:** Zhao Wang.
